# Remote ischemic conditioning reduces adverse events in patients with acute ischemic stroke complicating acute myocardial infarction: a randomized controlled trial

**DOI:** 10.1186/s13054-023-04786-y

**Published:** 2024-01-02

**Authors:** Sijie Li, Xiurong Xing, Lanjing Wang, Jiali Xu, Changhong Ren, Yalin Li, Jing Wang, Zhi Liu, Heng Zhao, Wenbo Zhao, Xunming Ji

**Affiliations:** 1 Department of Emergency, Xuanwu Hospital, Capital Medical University, Beijing, 100053 China; 2 Clinical Center for Combined Heart and Brain Disease, Capital Medical University, Beijing, 100069 China; 3 Beijing Institute of Brain Disorders, Collaborative Innovation Center for Brain Disorders, Capital Medical University, Beijing, 100069 China; 4 Department of Neurology, Xuanwu Hospital, Capital Medical University, No. 45, Changchun Street, Xicheng District, Beijing, 100053 China; 5 Beijing Key Laboratory of Hypoxic Conditioning Translational Medicine, Xuanwu Hospital, Capital Medical University, Beijing, 100053 China

**Keywords:** Acute ischemic stroke, Acute myocardial infarction, Major adverse cardiac and cerebrovascular events, Remote ischemic conditioning

## Abstract

**Background:**

Acute ischemic stroke (AIS) complicating an acute myocardial infarction (AMI) is not uncommon, but can severely worsen the clinical prognosis. This study aimed to investigate whether remote ischemic conditioning (RIC) could provide clinical benefits to patients with AIS complicating AMI.

**Methods:**

Subjects with AIS complicating AMI were recruited in this double-blind, randomized, controlled trial; assigned to the RIC and sham groups; and respectively underwent twice daily RIC and sham RIC for 2 weeks. All subjects received standard medical therapy. The primary endpoint was the rate of major adverse cardiac and cerebrovascular events (MACCEs) within 3 months after enrollment. MACCEs comprise of death from all causes, unstable anginas, AMI, acute ischemic strokes, and transient ischemic attacks.

**Results:**

Eighty subjects were randomly assigned; 37 patients in the RIC group and 40 patients in the sham-RIC group completed the 3-month follow-up and were included in the final analysis. Both RIC and sham RIC procedures were well tolerated. At 3-month follow-up, 11 subjects (29.7%) in the RIC group experienced MACCEs compared to 21 (52.5%) in the sham group (hazard ratio [HR], 0.396; 95% confidence interval, 0.187–0.838; adjusted *p* < 0.05). Six subjects (16.2%) in the RIC group had died at the 3-month follow up, significantly lower than the 15 (37.5%) deaths in the sham group (adjusted HR 0.333; 95% CI 0.126–0.881; *p* = 0.027). Seventeen subjects (45.9%) in the RIC group and 6 subjects (15.0%) in the sham group achieved functional independence (mRS score ≤ 2) at 3-month follow-up (adjusted OR 12.75; 95% CI 2.104–77.21; *p* = 0.006).

**Conclusions:**

Among patients with acute ischemic stroke complicating acute myocardial infarction, treatment with remote ischemic conditioning decreased the major adverse cardiac and cerebrovascular events and improved functional outcomes at 90 days.

*Trial registration:* URL: www.clinicaltrials.gov. Unique identifier: NCT03868007. Registered 8 March 2019.

## Introduction

Acute ischemic stroke (AIS) and acute myocardial infarction (AMI) are the two leading causes of mortality and disability worldwide, and these two acute, life-threatening syndromes share common risk factors and pathophysiological mechanisms. Thus, concurrence or one occurring after the other are not uncommon in clinical practice. It has been reported that approximately one in five AIS patients have significant cardiac events after AIS, and one in third of these events are AMI [1, 2]. The combination of these two acute events could further worsen the prognosis compared with each condition alone: it has been reported that in-hospital mortality of patients with both conditions is approximately 21–37%, and more than 56% of patients die within a year [3, 4].

Both AIS and AMI are emergencies that require timely reperfusion, but different treatment strategies are recommended [5]. Both conditions can be treated using intravenous thrombolysis with recombinant tissue plasminogen activator, but different dosages and time windows are recommended. In addition, although endovascular thrombectomy for AIS and percutaneous coronary intervention for AMI are recognized as effective management tools, the procedure order is controversial since performing one ahead of the other may lead to compromise of either the brain or the heart [6]. Furthermore, high doses of antithrombotic therapy are usually started after AMI, which may predispose patients with AIS to develop intracerebral hemorrhage, especially when patients undergo reperfusion therapy (such as intravenous thrombolysis and endovascular thrombectomy) [7]. Currently, the management of patients with AIS complicating AMI remains a great challenge for both stroke physicians and cardiologists.

Remote ischemic conditioning (RIC), a noninvasive strategy with one or more cycles of brief and transient limb ischemia, has been demonstrated to confer protection against prolonged and severe ischemia in distant organs (e.g., brain and heart) [8, 9]. It has been widely investigated in patients with AIS, and results showed that RIC can provide benefit patients with AIS by reducing the extent of brain tissue infarction and improving neurological outcomes [10, 11]. In addition, though recent large trials investigating the efficacy of RIC in the context of primary percutaneous intervention and cardiac surgery have proven predominantly neutral [12]. Previous basic research and proof-of-concept trials did confirm that RIC could reduce plasma myocardial enzyme levels, infarct volume, and the incidence of post-AMI heart failure in patients with AMI [13, 14]. The protective effects of RIC in reducing the infarct size AMI could thereby alleviate their severity and reducing related complications and other clinical events (including all causes of death), and previous studies have found that RIC could reduce the rate of major adverse cardiac and cerebral events (MACCEs) in patients undergoing elective percutaneous coronary intervention [15, 16].

Given these strong preliminary results in cardio-cerebrovascular diseases, and the promising data from proof-of-concept clinical trials, we hypothesized that RIC could provide benefits to patients with AIS complicating AMI. In this randomized clinical trial, we aimed to investigate whether two weeks of RIC treatment is safe and effective in patients with AIS complicating AMI.

## Methods

### Study design and participants

This randomized, double-blind, sham-controlled trial was conducted at Xuanwu Hospital, Capital Medical University. The trial was registered at www.clinicaltrials.gov (Unique identifier: NCT03868007), approved by the Ethics Committee of Xuanwu Hospital of Capital Medical University, and has been performed in accordance with the ethical principles of the Helsinki Declaration. Randomization was performed by opaque envelopes that concealed the group allocation. All participants or their legally authorized representatives provided informed consent before enrollment.

In this study, participants were consecutively recruited from the emergency department and stroke unit. All patients presented with neurological deficits and who were diagnosed with AIS within 24 h of symptom onset were evaluated. They were screened for inclusion if they did not receive reperfusion therapy. AIS was defined as a clinical episode of neurological dysfunction caused by focal cerebral infarction that could be detected on neuroimaging [17]. AMI was defined as a rise of plasma cardiac biomarkers (e.g., myocardial enzymes), along with supportive evidence in the form of typical symptoms (e.g., chest pain), suggestive electrocardiographic changes, or imaging evidence of the new loss of viable myocardium or new regional wall motion abnormalities [18]. The inclusion criteria were as follows: (1) age ≥ 60 years; (2) subjects with AIS complicated by AMI within 24 h of symptom onset; (3) tolerance to medications for the management of cardiocerebrovascular disease, including aspirin, clopidogrel, and statins; (4) stable vital signs, and normal renal and hepatic functions; (5) informed consent provided by the subjects or their legally authorized representative.

The exclusion criteria were: (1) patients with AMI undergoing percutaneous coronary intervention; (2) patients with AIS undergoing intravenous thrombolysis or endovascular thrombectomy; (3) any disorder that could potentially increase pre-stroke myocardial enzyme concentrations; (4) coronary artery stenosis requiring coronary bypass surgery for the index event within three months; (5) severe heart failure requiring mechanical ventilation or use of an intra-aortic balloon pump; (6) pre-stroke modified Rankin scale score ≥ 2; (7) uncontrolled hypertension (defined as systolic blood pressure ≥ 200 mmHg despite medications at enrollment); (8) any vascular, soft tissue, or orthopedic injury that were contraindications for RIC or sham RIC procedures; (9) upper limbs artery or subclavian artery stenosis > 70%.

### Interventions

Upon admission, all patients with AIS were initially evaluated by a stroke neurologist and underwent routine electrocardiogram testing. If the electrocardiogram results indicated AMI or the patients presented with symptoms of AMI, plasma myocardial enzyme concentrations were measured, and a cardiologist was consulted. Eligible subjects with AIS-complicating AMI received medical management, including antiplatelet therapy, statins, and management for cardiocerebrovascular risk factors. Administration of antihypertensive, antidiabetic, or other agents was at the treating physician's discretion based on the individual’s conditions. Additionally, subjects in the RIC and sham RIC groups underwent RIC or sham RIC procedures twice daily for 14 consecutive days.

Randomized controlled trials were conducted using RIC and sham RIC procedures. Both procedures were performed immediately after enrollment and within 24 h of stroke onset. These procedures involved five cycles of simultaneous bilateral arms ischemia for 5 min, followed by reperfusion for another 5 min, with one RIC procedure requiring 45 min. RIC and sham RIC procedures were performed using identical electric auto-control devices. Still, the cuff pressures during the ischemia period were different, with 200 mmHg and 60 mmHg for RIC and sham RIC procedures, respectively. A trained nurse assisted with these procedures during hospitalization. All patients and medical staff were unaware of the assigned treatment.

### Safety assessment

Safety outcomes were objective signs of upper limb injury that including local edema, petechia, ecchymosis, or skin lesions, and any other adverse events related to RIC.

### Efficacy assessment

The primary efficacy endpoint of this study was the rate of MACCEs within 3 months after enrollment, which comprises death from all causes, unstable angina, AMI, AIS, and transient ischemic attack.

The secondary efficacy endpoints of this study included (1) the proportion of subjects who achieved functional independence (defined as modified Rankin Scale [mRS] ≤ 2 points) at 3-month follow-up; and (2) changes in mRS scores.

### Statistical analysis

Sample size and power were calculated using PASS 11 based on previous studies [3, 19, 20]. MACCEs within three months after the index stroke was expected to occur in 72% of subjects in the sham group, and this rate was expected to be reduced to 40% in the RIC group [16]. The intended target sample size was approximately 80 subjects (40 in each group), allowing for a 5% loss to follow-up at three months, 80% power, and an alpha significance value of 0.05 (two-sided).

Statistical analyses were performed with SPSS (version 21.0, IBM Inc.), and survival curves, distributions of 3-month mRS, and changes in mRS scores and NIHSS scores were derived using GraphPad Prism 6 (GraphPad Software, La Jolla, CA). Continuous variables were described as mean ± SD or medians (interquartile range, IQR), and t-tests or Mann–Whitney *U* tests were performed to detect between-group differences. Binary data were summarized as frequencies and percentages, and between-group comparisons were performed via the *χ*^2^ test or Fisher’s exact tests. Kaplan–Meier (KM) survival analysis was used to estimate the risk of the primary endpoint. Effects of RIC on endpoints were separately adjusted by the Cox proportional hazard model using hazard ratios (HR) and 95% confidence intervals (CI). A *p* value of ≤ 0.05 (two-sided) was considered significant for all analyses.

## Results

### Baseline characteristics

**Fig. 1:**
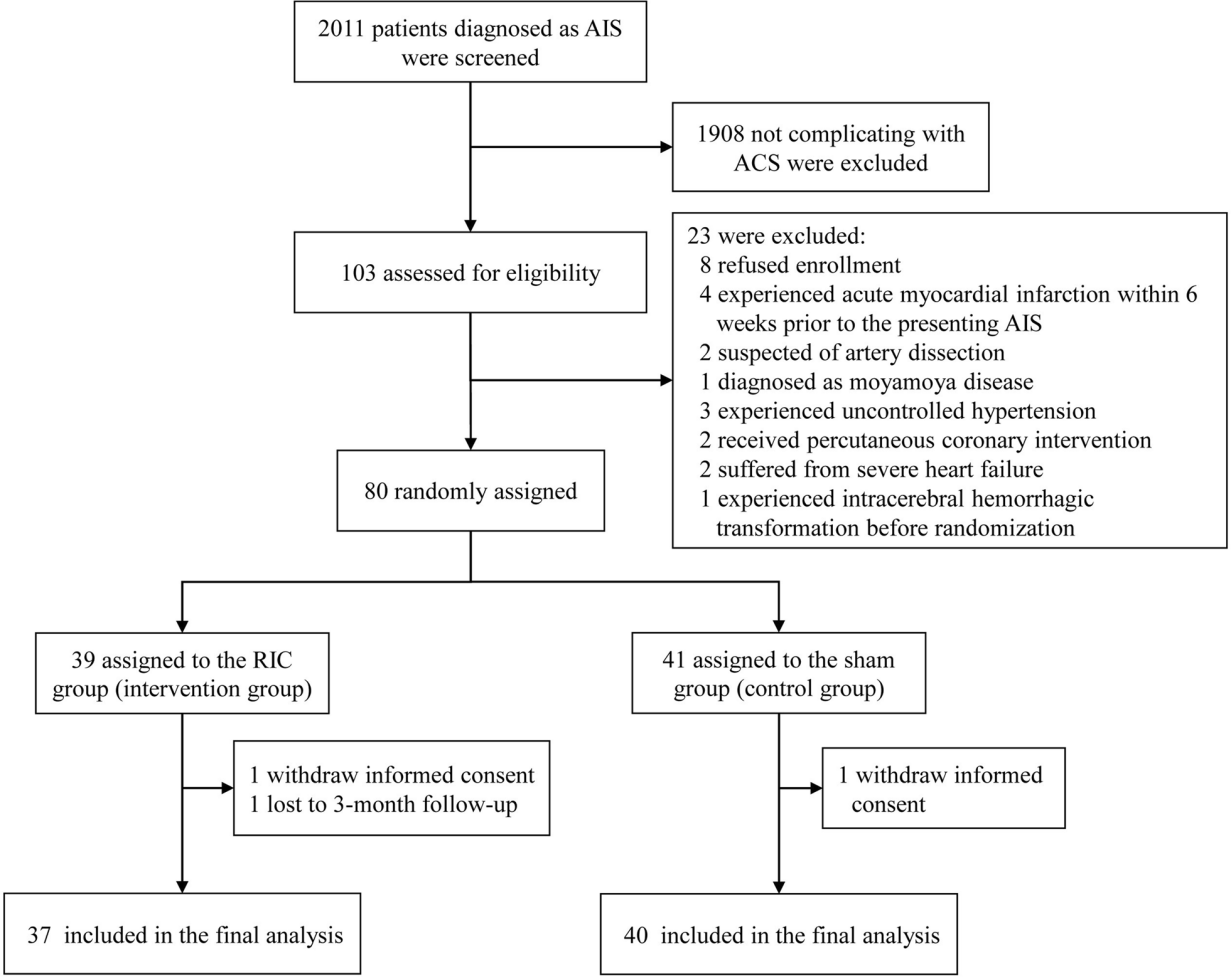
Enrollment and randomization. *AIS* acute ischemic stroke, *AMI* acute myocardial infarction, *RIC* remote ischemic conditioning

Between March 10, 2019, and April 10, 2022, a total of 2011 patients with AIS who did not receive reperfusion therapy were screened for eligibility. In total, 103 subjects with complicating AMI were assessed for eligibility, and 80 were randomly allocated; 39 subjects were included in the RIC group and 41 in the sham RIC group. The flowchart of this study is summarized in the Fig. 1, and reasons for excluding 23 patients were also included. A total of 37 subjects in the RIC group and 40 in the sham group were included in the final analysis.

The baseline characteristics of the two groups are summarized in Table 1. The average age was 74.97 ± 11.55 years for the RIC group and 74.75 ± 11.58 years for the sham group (*p* = 0.993). The etiology of AIS in each group is clarified based on the TOAST criteria, the most frequently used tool to establish ischemic stroke etiology, the proportions of large artery atherosclerosis are 64.9% in the RIC group and 65.0% in the sham RIC group. In addition, the proportion of AIS secondary to intracranial artery stenosis are 48.7% in the RIC group and 45% in the sham RIC group. Other baseline characteristics, including NIHSS scores, blood pressure, vascular risk factors, GRACE scores, and pre-stroke antithrombotic therapy, are also summarized in Table 1, and no between-group differences were detected.

**Table 1:** Baseline characteristics of AIS patients complicating AMI

Characteristic	RIC (*N* = 37)		Sham-RIC (*N* = 40)	*p* value
Age—yr	74.97 ± 11.55		74.75 ± 11.58	0.933
Male sex—no. (%)	23 (62.2%)		24 (60.0%)	0.846
Admission SBP, mmHg	130.49 ± 27.93		130.40 ± 25.68	0.989
Admission DBP, mmHg	74.16 ± 15.54		71.80 ± 16.13	0.515
Serum glucose, mmol/L	7.48 ± 2.78		8.50 ± 4.44	0.233
LDL, mmol/L	2.36 ± 0.86		2.43 ± 1.13	0.738
hs-CRP, mg/L	16.20 (8.21–30.50)		30.50 (5.95–40.54)	0.230
*Vascular risk factor*				
Hypertension, *n* (%)	30 (81.1%)		25 (62.5%)	0.071
Diabetes, *n* (%)	13 (35.1%)		15 (37.5%)	0.829
Hyperlipidemia, *n* (%)	9 (24.3%)		12 (30.0%)	0.576
Arrhythmia, *n* (%)	13 (35.1%)		16 (40%)	0.660
Atrial fibrillation, *n* (%)	7 (18.9%)		10 (25%)	0.520
Smoke, *n* (%)	10 (27.0%)		15 (37.5%)	0.327
Alcohol, *n* (%)	4 (10.8%)		6 (15%)	0.585
History of prior coronary heart disease, *n* (%)	20 (54.1%)		17 (42.5%)	0.311
Prior ischemic stroke, *n* (%)	15 (40.5%)		19 (47.5%)	0.539
*Pre-stroke antithrombotic therapy*				
None, *n* (%)	9 (24.3%)		11 (27.5%)	0.751
Single antiplatelet, *n* (%)	25 (67.6%)		29 (72.5%)	0.637
Dual antiplatelet, *n* (%)	3 (8.1%)		0 (0.0%)	0.106
*Location of AIS*				0.818
Anterior circulation, *n* (%)	25 (67.6%)		28 (70.0%)	
Posterior circulation, *n* (%)	12 (32.4%)		12 (30.0%)	
Stroke etiology*				0.884
Large-artery atherosclerosis, *n* (%)	24 (64.9%)		26 (65.0%)	
Cardioembolism, *n* (%)	5 (13.5%)		6 (15.0%)	
Small-vessel occlusion, *n* (%)	5 (13.5%)		4 (10.0%)	
Stroke of other determined etiology, *n* (%)	0 (0%)		1 (2.5%)	
Stroke of undetermined etiology, *n* (%)	3 (8.1%)		3 (7.5%)	
Intracranial artery stenosis, *n* (%)^^^	18 (48.7%)		18 (45%)	0.749
*Acute coronary syndrome type*				0.642
STEMI, *n* (%)	6 (16.2%)		5 (12.5%)	
NSTEMI, *n* (%)	31 (83.8%)		35 (87.5%)	
*Severity of AMI*				
GRACE score	177.0 (160.0–194.5)		190.5 (163.3–206.5)	0.155
*Severity of stroke*				
NHISS score	8 (4–12)		6.25 (4.25–14)	0.810
mRS score	4 (3.5–5)		4 (4–4)	0.787
LVEF, %	63.0 (49.0–67.0)		57.1 (51.0–66.0)	0.588
Stroke onset to enrollment, h	17.00 (13.00–20.00)		19.75 (12.00–22.38)	0.232
Stroke onset to RIC treatment, h	18.00 (14.00–21.50)		21.00 (13.50–23.60)	0.245

*RIC* remote ischemic conditioning, *SBP* systolic blood pressure, *DBP* diastolic blood pressure, *LDL* low-density lipoprotein, *hs-CRP* high-sensitivity C-reactive protein, *AIS* acute ischemic stroke, *AMI* acute myocardial infarction, *STEMI* segment elevation myocardial infarction, *NSTEMI* non-segment elevation myocardial infarction, *NIHSS* National Institutes of Health Stroke Scale, *mRS* modified Rankin Scale, *LVEF* Left ventricular ejection fraction

*Clarified based on TOAST criteria

^Refer to intracranial artery stenosis that cause the indexed ischemic stroke event

### Safety and feasibility

Both RIC and sham RIC procedures were well tolerated by all subjects, and no serious adverse events related to the procedures were reported. One subject in the RIC group experienced visible petechiae in bilateral upper limbs, which resolved before the next training session. Another subject in the RIC group reported feeling dizzy and chest stuffiness during the first cycle of cuff inflation, but the symptoms resolved after cuff deflation, and all subsequent RIC cycles were completed without any similar symptoms. Totally, two subjects (5.4%) experienced related adverse events in the RIC group as compared with none in the sham group (*p* = 0.23).

### MACCEs

**Fig. 2:**
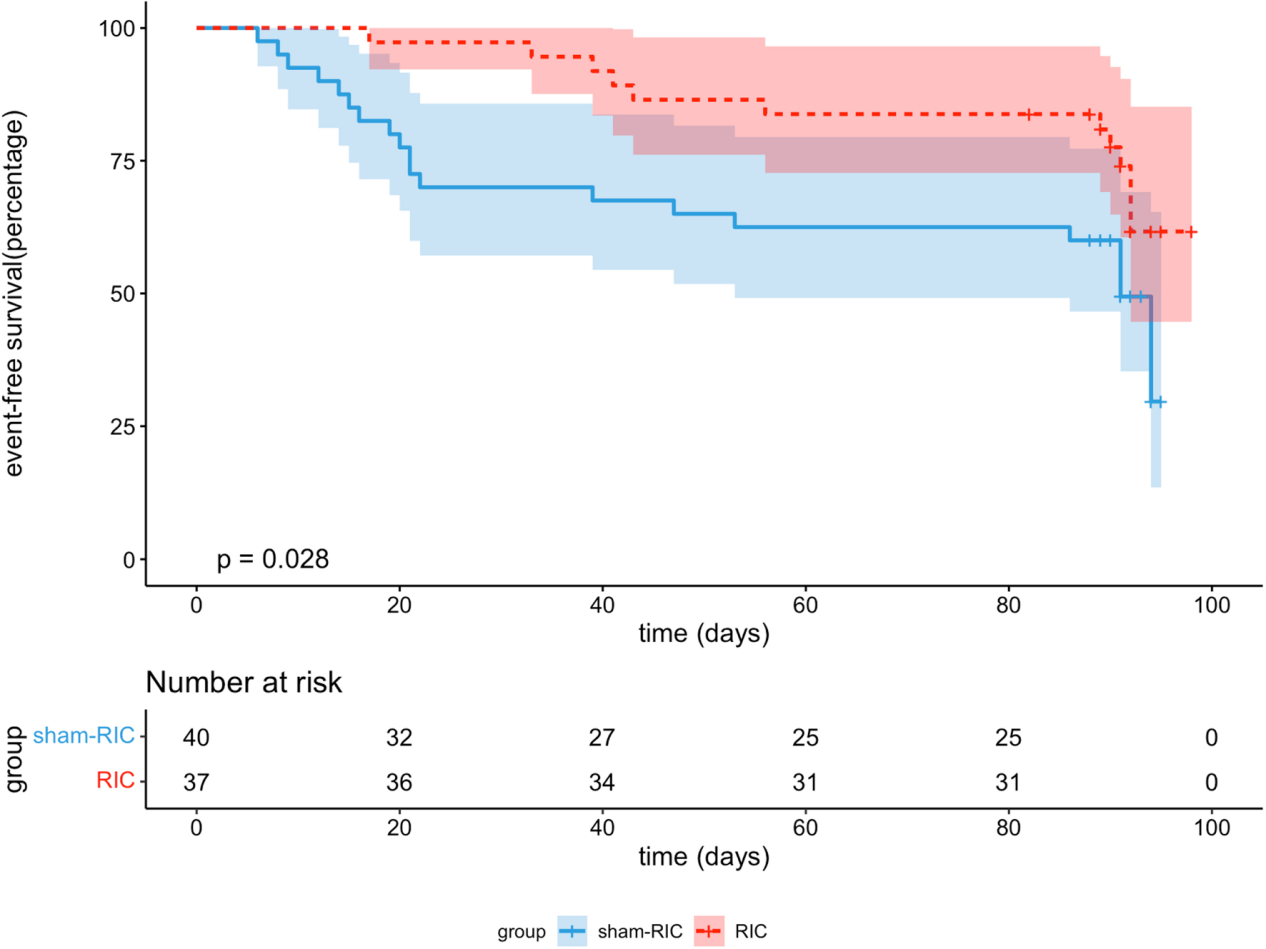
Kaplan–Meier event curve for the primary endpoint. Cumulative incidence of major adverse cardiac and cerebrovascular events (a comprise death from all causes, unstable angina, acute myocardial infarction, acute ischemic stroke, and transient ischemic attack)

At 3-month follow-up, 11 subjects (29.7%) in the RIC group and 21 subjects (52.5%) in the sham group experienced MACCE (HR 0.442, 95% CI 0.213–0.918, *p* = 0.029). After adjusting for potential confounders, the incidence of MACCEs remained lower in the RIC group compared with the sham-RIC group (adjusted HR 0.396, 95% CI 0.187–0.838, *p* = 0.015) (Table 2). The KM survival analysis also showed that the RIC group had a lower risk of MACCEs (Fig. 2, p = 0.028). Six subjects (16.2%) in the RIC group had died, significantly lower than the 15 subjects (37.5%) in the sham group (adjusted HR 0.333, 95% CI 0.126–0.881, *p* = 0.027). There was no significant difference between the two groups regarding the incidence of AIS and AMI.

**Table 2:** Clinical and functional outcomes of all subjects

Clinical outcomes	RIC (*N* = 37)	Sham-RIC (*N* = 40)	HR/OR*	*p* value	Adjusted HR/OR*		Adjusted *p*
Primary outcome—no. (%)	11 (29.7%)	21 (52.5%)	0.442 (0.213–0.918)	0.029	0.396 (0.187–0.838)^a^		0.015
All-cause death	6 (16.2%)	15 (37.5%)	0.341 (0.132–0.880)	0.026	0.333 (0.126–0.881)^a^		0.027
Recurrence of AIS	1 (2.7%)	5 (12.5%)	0.160 (0.019–1.373)	0.095	0.116 (0.012–1.124)^a^		0.063
Recurrence of AMI	10 (27.0%)	16 (40.0%)	0.516 (0.234–1.138)	0.101	0.483 (0.216–1.018)^a^		0.076
3-month functional independence	17 (45.9%)	6 (15.0%)	4.675 (1.582–13.819)	0.005	12.75 (2.104–77.21)^b^		0.006
*mRSscore*							
1 month	3 (3–4)	4 (3–4)	–	0.002			
3 month	3 (2–4)	4 (3–4)	–	0.004			
*NIHSS*							
1 week	3 (0–0.75)	6.5 (2.25–11.75)	–	0.020	–		
2 weeks^†^	2 (0–5)	6 (1–11)	–	0.012	–		

*RIC* remote ischemic conditioning, *HR* hazard ratio, *OR* odd ratio, *AIS* acute ischemic stroke, *AMI* acute myocardial infarction, *NIHSS* National Institutes of Health Stroke Scale, *mRS* modified Rankin Scale

^a^Adjusted for age, admission systolic blood pressure, baseline GRACE score

^b^Adjusted for age, sex, admission NHISS score, baseline Grace score.

*The effect of RIC on primary outcomes was depicted as HR, and the effect on 3-month functional independence was shown as OR

^†^Four patients suffered death in sham-RIC group within two weeks who were excluded in this analysis

### Functional outcomes

**Fig. 3:**
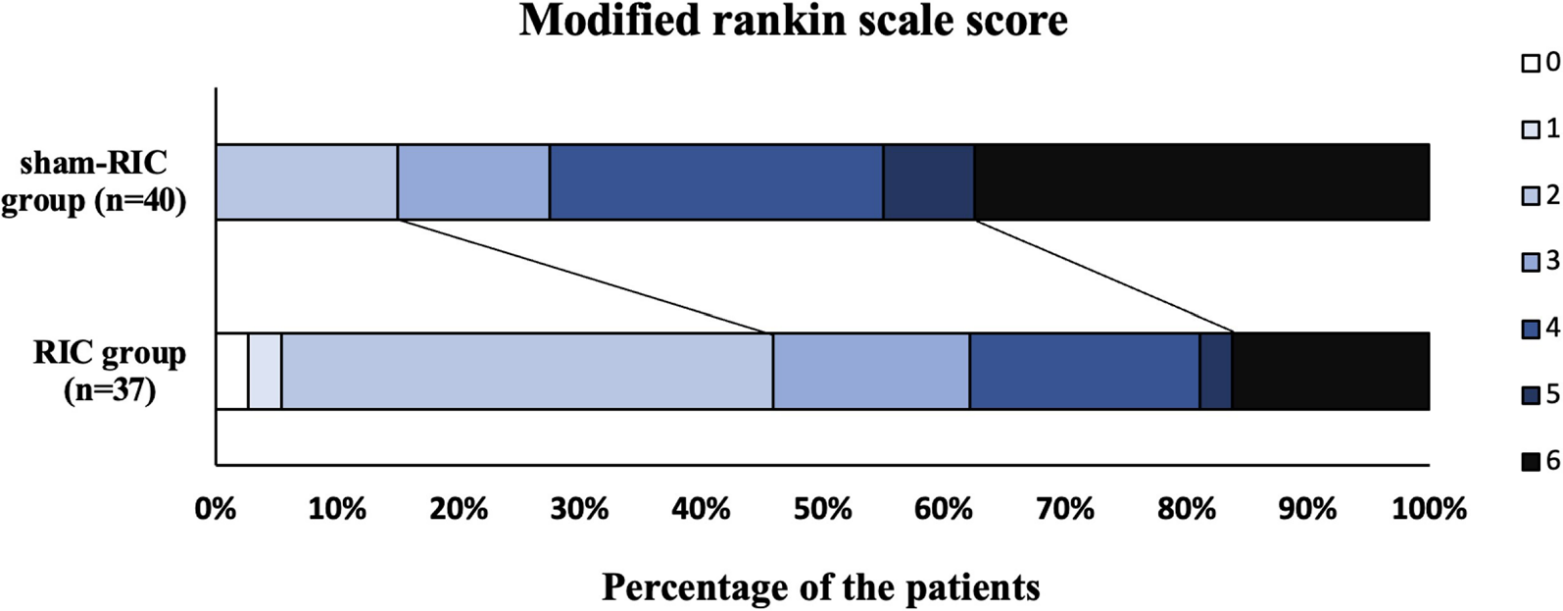
Distributions of functional scores at 3 months. RIC, remote ischemic conditioning. Showed are the scores of the mRS for all subjects

The distribution of mRS scores is illustrated in the Fig. 3. At 3-month follow-up, 17 subjects (45.9%) in the RIC group and six subjects (15.0%) in the sham group achieved functional independence (mRS score ≤ 2), and there were significant differences between groups (adjusted OR 12.75, 95% CI 2.104–77.21, *p* = 0.006). The median mRS score was 3 (IQR 2–4) in the RIC group compared with 4 (IQR 3–4) in the sham group (*p* < 0.05).

## Discussion

In this study, we found that in patients with AIS complicating AMI within 24 h of symptom onset, two weeks of RIC treatment was safe, and it decreased the composite clinical outcomes of mortality and cardiocerebrovascular events and improve the proportion of functional independence at 3-month follow-up.

In patients with AIS, RIC has been demonstrated to improve 3-month functional outcomes regardless of whether they previously received intravenous thrombolysis [10, 11]. Consistent with previous studies, this study also found that RIC could improve functional outcomes if initiated during the acute phase and used consecutively for two weeks. In patients with AMI, previous studies found that RIC could reduce myocardial injury, but could not provide clinical benefits [21–23], which was inconsistent with this study. The discrepancy may be explained by differences study populations, which may be easier to get positive results. In this study, elderly patients with AIS-complicating AMI were recruited, and this population often has more severe conditions than patients undergoing elective cardiac surgery or patients with ST-elevation myocardial infarction undergoing percutaneous coronary intervention [24]. The incidence of 3-month MACCEs was over 50% in this study, whereas the incidence of 1-year MACCEs in previous studies was less than 30%.

This study has several limitations. First, the RIC treatment protocol used in this study was rather pragmatic and tailored to subjects with AIS-complicating AMI. Second, due to the inherent transient paroxysmal attack characteristics of TIA and angina, the possibility of missing adverse events could be completely ruled out, and clinical outcomes may have been underestimated, causing potential bias. Third, as it is difficult to determine the onset time orders of AIS and asymptomatic AMI, and there might be patients who had presented with their stroke but had their AMI over 24-h prior to AIS enrolled into this study. In addition, the underlying mechanisms of such cardio-cerebral protection were not investigated.

## Conclusions

In conclusion, in elderly patients with AIS complicating AMI, RIC is a safe and effective therapy to reduce 3-month adverse clinical outcomes if initiated within 24 h of onset and performed twice daily for two weeks. These findings warrant a large multicenter randomized controlled phase 3 trial to confirm the efficacy of RIC, and the underlying mechanisms also need further investigation.

## Data Availability

The datasets used during the current study are available from the corresponding author on reasonable request.

## Abbreviations

AIS: Acute ischemic stroke
AMI: Acute myocardial infarction
RIC: Remote ischemic conditioning
MACCE: Major adverse cardiac and cerebrovascular event
mRS: Modified Rankin Scale
KM: Kaplan–Meier
HR: Hazard ratio
CI: Confidence interval
